# Kazakhstan gears up to launch social health insurance 

**DOI:** 10.2471/BLT.16.031116

**Published:** 2016-11-01

## Abstract

Twenty years ago Yelzhan Birtanov was a physician working in one of Kazakhstan’s hospitals, today he is helping to roll out universal coverage of health services in the Central Asian country. He talks to Vijay Shankar Balakrishnan.

Q: How did you become interested in health care?

A: My parents were physicians and I grew up in a medical atmosphere. While I studied medicine I also worked as a junior nurse in a hospital doing night shifts. Before graduating in 1994, I visited the University Medical Centre of Arizona University in Tucson, in the United States of America. I was impressed to see one of the most advanced health-care systems and was painfully aware of the stark differences between this and the health care in my country at that time.

Q: How well could the Kazakh people access health care during the transition to the market economy after the end of the Soviet Union? 

A: The first years after independence in 1991 were the hardest. I was working in a hospital at the time and remember the shortages of medicines, medical supplies and devices. Patients often had to bring their own medicines, food and blankets to hospital and pay under the counter for services. Physicians’ salaries were very low and much of the financial burden was on the patients. It was difficult for the government to maintain a network of primary care centres and hospitals providing health care and to pay health workers adequately because of our financial difficulties due to the abrupt transition to a market economy. Many nurses and doctors felt demoralized and left the health-care system. 

Q: The declaration of Health for all was adopted in Alma-Ata, the former Kazakh capital, in 1978 by WHO’s Member States. Yet primary health care was largely undeveloped in Kazakhstan and many other parts of the former Soviet Union before it broke up in 1991. How did the Kazakh health system evolve after that? 

A: We tried different financing models before starting to roll out universal coverage of health services from about 2001. The government wanted to provide all levels of care, from primary care, immunization, to secondary and tertiary care. But it was difficult and many services were not included. Under the first national health programmes that were rolled out from 2005–10, we increased the number of general practitioners and primary health care centres and introduced new health-care services, such as check-ups for cardiovascular diseases at primary care level, and we increased efforts to encourage evidence-based practice among health-care providers. A unified health information system was established in hospitals and health clinics throughout the whole country. In the next national health programme for the years 2011–2015, we expanded some services, such as screening for noncommunicable diseases, and we increased the number of general practitioners by 30%. 

Q: How did you finance these recent reforms? 

A: We introduced new payment methods and financial incentives, such as capitation at primary health care level (a system whereby general practitioners are allocated a set sum for each head of population assigned to them) and diagnosis-related group payment methods for hospitals and clinics (a system for classifying health-care services for health insurance reimbursement purposes). In 2014, we introduced the capitation payment method which allowed us to increase health-care financing at primary health care level. Overall health-care spending has increased 1.5 times: from US$ 1.69 billion (562.8 billion tenge) in 2010 to US$ 2.61 in 2014.

*Q: In 2008, primary health received only 16% of the national health budget compared with 53.4% for inpatient care. How much has primary health care provision increased in recent years*? 

A: The demand for inpatient services has declined because many patients can now see a general practitioner. The average hospital stay is 27% shorter than it was in 2010 and the number of patients seen at primary care level has increased by 23.5% since 2010. The 2016–19 national health programme, *Densaulyk*, which means “health” in Kazakh, also seeks to bolster primary health care. The first goal is to implement public health policy incentives to prevent and manage diseases at primary care level, the second is to achieve a more effective and financially stable health-care system and the third is to introduce mandatory social health insurance next year. 

Q: What are your priorities in the provision of primary health care? 

A: The biggest health burden is of heart disease, cancer and other noncommunicable diseases (NCDs). That’s why we are developing our health system infrastructure to improve the health-care management and financing, and health promotion and protection. Over the next three years, we will be integrating the prevention and control of NCDs further into primary health care. To achieve that we need to increase these services at primary care level and caregivers from different disciplines need to work more closely together. By 2017, all parts of the country will be providing disease management for hypertension, diabetes and heart disease and the prevention of noncommunicable diseases at primary level. 

*Q: As noted by *Health systems in transition:**Kazakhstan*, 7.4% of the population in 2008 did not use health services because of the high costs. Are patients paying less out-of-pocket now?*

A: It’s difficult to say, as we did not measure out-of-pocket payments before 2005. Currently we are still seeing a high level of such expenditure, 35.4%, according to data from our national health accounts system. There are still some cases of unofficial payments for health-care services and patients still have to make out-of-pocket payments because not all health-care services and pharmaceutical products are subsidized. We are not there yet. 

Q: Will the new social health insurance scheme address this?

A: We hope so. Our vision is one of sustainable financing for our health-care system which ensures adequate allocation of funds, their equitable distribution and efficient use. To achieve this, our population benefits from a guaranteed package of free health services that is funded by the government budget. This will cover emergency cases, prevention and treatment for certain diseases, such as HIV infection and tuberculosis, and vaccinations. In addition, other forms of health care will be covered by a new mandatory social health insurance system, covering other primary, ambulatory and inpatient services, and medicines.

Q: Medicines are not always subsidized and can be very expensive for many people in countries of the former Soviet Union. Will medicines now be provided free of charge in Kazakhstan? 

A: We have a list of about 400 medicines to be provided free of charge covering many clinical areas, including oncology, cardiovascular, gastrointestinal, respiratory and psychiatric conditions. These will be covered by the mandatory social health insurance system that we are launching next year. Under the new scheme, funds will be pooled from three sources: the government (to cover the socially vulnerable, such as children, the elderly, pregnant women and the unemployed), as well as contributions from employers and employees. 

Q: As mentioned you lost many health-care professionals earlier, are any of them returning to Kazakhstan. How are you retaining health-care professionals in the new reform? 

A: It’s difficult to say whether health professionals are coming home, but we hope to retain the new generation of health workers better with these new initiatives and that it will become more attractive to work in our health system. We are reviewing the structure of human resources in our national health-care system and we have been investing in medical training since 2005. We have six medical schools, which have three-to-five-year agreements with selected training colleges around the world and some of our trainee physicians, health-care managers and nurses are sent to partner countries, including Germany, Israel, the Russian Federation and the United Kingdom of Great Britain and Northern Ireland for further training. 

Q: What are your targets for shifting much of the health care burden from hospitals to primary health care physicians and family physicians? 

A: As the number of general practitioners increases, they will replace district hospital physicians. The idea is that general practitioners should cover a wide range of services including family health protection, family planning, disease prevention and treatment and one of the priorities for primary care will be strengthening maternal and child health care at primary care level. Our key target indicators include increasing primary care financing to 40% of the total expenditure on health by 2019 (from 28% currently), increasing the number of general practitioners to 26.6% of the total number of physicians by 2019 (from 21% currently), decreasing the number of people served by one general practitioner to 1577 from the current level of 2194 by 2019 and increasing the number of primary care facilities to 6.5 facilities per 10 000 population by 2019 (from 4.5 currently). 

Q: A recent paper published in BMJ Global Health highlighted weaknesses in Kazakhstan’s health monitoring. How will you monitor and assess your performance in coming years, so that you will know whether your country has met its health goals or not?

A: We often suffer from a lack of strong monitoring and evaluation. We have comprehensive strategies, but implementing and monitoring can be difficult. We need to separate these into political monitoring within the government, by governmental institutions, and scientific and public monitoring, by research institutions. We also plan to involve more nongovernmental organizations and professional associations in our monitoring systems to produce a more independent assessment of our health-care programmes.

Q: What are your key health goals in terms of life expectancy, maternal and child health? 

A: The current 2011–2015 programme has achieved good results. We were able to increase average life expectancy from 68.45 years in 2010 to 71.7 today and to almost halve child mortality (from 16.58 deaths of children younger than five years per 10 000 live births in 2010 to 9.7 in 2015) and to halve maternal mortality (from 22.7 deaths per 100 000 live births in 2010 to 11.7 in 2015). We aim for further improvements by 2019. Our goals are to increase average life expectancy from 71.7 years in 2015 to 73 years, to decrease child mortality from 9.7 deaths of children younger than five years per 10 000 live births to 9.1 in 2015 and to reduce maternal mortality from 11.6 deaths in 2015 to 11.2 per 100 000. 

Q: What health achievements are you most proud of? 

A: During a short period of time we have been able to increase average life expectancy of our population, decrease maternal and child mortality, and decrease the prevalence of tuberculosis and reduce tuberculosis mortality. We aim to achieve the life expectancy and child survival levels of the world’s most developed countries. But we still have a long way to go.

